# Predicting Acute Kidney Injury after Cardiac Surgery by Machine Learning Approaches

**DOI:** 10.3390/jcm9061767

**Published:** 2020-06-07

**Authors:** Charat Thongprayoon, Panupong Hansrivijit, Tarun Bathini, Saraschandra Vallabhajosyula, Poemlarp Mekraksakit, Wisit Kaewput, Wisit Cheungpasitporn

**Affiliations:** 1 Division of Nephrology, Department of Medicine, Mayo Clinic, Rochester, MN 55905, USA; charat.thongprayoon@gmail.com; 2 Department of Internal Medicine, University of Pittsburgh Medical Center Pinnacle, Harrisburg, PA 17105, USA; hansrivijitp@upmc.edu; 3 Department of Internal Medicine, University of Arizona, Tucson, AZ 85724, USA; tarunjacobb@gmail.com; 4 Department of Cardiovascular Medicine, Mayo Clinic, Rochester, MN 55905, USA; Vallabhajosyula.Saraschandra@mayo.edu; 5 Department of Internal Medicine, Texas Tech University Health Sciences Center, Lubbock, TX 79424, USA; poemlarp@gmail.com; 6 Department of Military and Community Medicine, Phramongkutklao College of Medicine, Bangkok 10400, Thailand; wisitnephro@gmail.com; 7 Division of Nephrology, Department of Medicine, University of Mississippi Medical Center, Jackson, MS 39216, USA

**Keywords:** acute kidney injury, AKI, cardiac surgery, machine learning, artificial intelligence, nephrology

## Abstract

Cardiac surgery-associated AKI (CSA-AKI) is common after cardiac surgery and has an adverse impact on short- and long-term mortality. Early identification of patients at high risk of CSA-AKI by applying risk prediction models allows clinicians to closely monitor these patients and initiate effective preventive and therapeutic approaches to lessen the incidence of AKI. Several risk prediction models and risk assessment scores have been developed for CSA-AKI. However, the definition of AKI and the variables utilized in these risk scores differ, making general utility complex. Recently, the utility of artificial intelligence coupled with machine learning, has generated much interest and many studies in clinical medicine, including CSA-AKI. In this article, we discussed the evolution of models established by machine learning approaches to predict CSA-AKI.

## 1. Introduction

Acute kidney injury (AKI) is a common and serious complication after cardiac surgery related to a complex set of exposures, including cardiopulmonary bypass, tissue damage, cardiac dysfunction, and hemolysis [1,2,3,4,5]. Depending on the definition, patient characteristics, and the type of cardiac surgery, the incidence of cardiac surgery-associated AKI (CSA-AKI) varies between 7% and 40%, Table 1 [6,7,8,9,10,11,12]. The incremental index hospitalization cost associated with CSA-AKI is higher than USD 1 billion in the United States [13]. The development of CSA-AKI has a dramatic impact on intensive care unit (ICU) and hospital length of stay as well as on short- and long-term mortality [14,15,16,17,18,19]. In patients with CSA-AKI requiring dialysis, mortality (at hospital discharge or 30-day mortality) can be as high as 60% to 70% [20]. Risk of end-stage kidney disease (ESKD) after cardiac surgery is also substantial, especially in patients with Acute Kidney Injury Network (AKIN) stage 2 or 3 AKI, with a hazard ratio of 3.8 to develop ESKD compared with all patients [21].

Several risk factors have been identified that are associated with an increased risk to develop CSA-AKI, including female sex, advanced age, left ventricular ejection fraction less than 35%, comorbidities (diabetes, hypertension, hypercholesterolemia, peripheral vascular disease, chronic obstructive pulmonary disease, congestive heart failure), preexisting chronic kidney disease (CKD), previous cardiac surgery, intraoperative (use of an intra-aortic balloon pump, more extended cardiopulmonary bypass (CPB) and prolonged aortic cross-clamping), severe bleeding requiring transfusion of blood products, a requirement for potent vasopressors, prolonged hypotension, and low cardiac output syndrome, systemic inflammatory response syndrome, more complex cardiac disease such as left main coronary disease, complex cardiac operations, and emergency surgery. Perioperative administration of nephrotoxic agents, such as angiotensin-converting enzyme inhibitors, aminoglycoside antibiotics, loop diuretics, or contrast media, may increase the risk of developing a CSA-AKI, Table 2 [20,45,46,47]. 

Attempts to improve AKI’s clinical outcomes have centered on early diagnosis and customized treatment [52]. Early identification of patients at high risk of CSA-AKI by applying risk prediction models allows clinicians to closely monitor these patients and start effective preventive and therapeutic approaches to lessen the incidence of AKI. Several AKI risk prediction models have been developed [14,16,17,53,54,55,56,57,58,59]. Risk assessment scores have been developed for CSA-AKI [14,16,17,53,54,55,56,57,58,59,60]. The Thakar Score [53], Mehta score [14], and Simplified Renal Index score [16] have been validated for predicting severe AKI that requires renal replacement therapy. The Thakar model has been examined comprehensively and found to have great discriminations. Nonetheless, dialysis events are uncommon, restricting the utility of these risk scores to prognosticate patients who do not need renal replacement therapy. Moreover, the definition of AKI and the variables utilized in these risk scores differ, making general utility complex [55]. Most of these risk scores are based on clinical factors that are accessible in the preoperative setting. These are helpful for risk stratification and counseling of patients. Nonetheless, perioperative events, including prolonged bypass, blood loss, and transfusion, can negatively influence the risk for CSA-AKI.

## 2. Predicting CSA-AKI by Machine Learning

The model established by machine learning approaches can effectuate early dynamic monitoring based on the actual objective data of all patients and conserve the time of clinicians. [61,62,63,64]. The rise of machine learning is driven by the ability to process “big data” and the need to deliver the best possible value- and evidence-based care. The utility of artificial intelligence (AI) coupled with machine learning, has generated much interest and many studies in clinical medicine [61,65,66,67,68,69,70,71,72,73,74,75,76,77,78,79]. The machine learning approach has been developed recently for advantages in performance and extensibility and has become indispensable for solving complex problems in most sciences [80,81,82]. This method is used to examine postoperative outcomes [83,84,85,86] and predict hypotension [87,88] and the depth of anesthesia [89,90,91,92,93,94]. Machine learning has also been applied in the fields of intensive care unit medicine [95], emergency medicine [96], and neuroimaging [97]. 

With the notable extension of the application of electronic health records (EHRs) in the area of big data [98,99,100], a substantial amount of EHR data and machine learning algorithms have advanced to fulfill an essential role in the clinical study of AKI. It is presently a relevant tool for AKI diagnosis and prediction [64]. The establishment of an AI-based clinical decision support systems (CDSS) based on a self-learning predictive model may be utilized for monitoring AKI among hospitalized patients in prospective clinical practice [101]. Compared with conventional analysis methods, recent studies have suggested that some machine learning algorithms may reach greater accuracy than the conventional logistic regression models [75,102,103]. Studies have shown that machine learning can predict AKI after general surgery, liver transplant, cardiac surgery, hepatectomy, severe burns, sepsis, and percutaneous coronary intervention [75,104,105,106,107,108,109,110]. Utilizing data from more than 700,000 subjects from multi-centers and stratified by an interval window of 6 hours, a recurrent neural network-based risk prediction model for AKI (AUC of 0.92] was verified [111]. AKI episodes were prognosticated within a 48-h window. Nevertheless, the area under the precision-recall curve was only 30%, which depicts a ratio of two false alerts for each actual alert [111]. 

Some machine learning algorithms have also been labeled as a “black box”, where there is limited insight into how the model is basing its prediction [112]. This draws into inquiry how clinicians can mitigate certain risk factors in patients to make them a more suitable candidate for treatment without knowing what is influencing their outcome. Nevertheless, there are some machine learning algorithms, such as XGBoost (eXtreme Gradient Boosting), where the relative magnitude of variables in prognosticating a particular outcome can be computed and envisioned. This renders a level of insight comparable to a logistic regression model about individual risk factors and their prognostic significance [113]. A gradient boosting machine (GBM) is currently a widespread approach for predicting AKI onset [75,105,107,114]. Huang et al. [107] presented a hazard prediction model for AKI following a percutaneous coronary intervention (PCI) based on GBM. The investigation involved a substantial amount of data from 947,091 cases that underwent PCI to set a baseline model. Besides, temporal validation was conveyed with data from greater than 900,000 hospitalized patients. The AUC of the GBM model was 79% greater than the baseline linear regression model. Recently, Lee et al. [75,105] presented a prediction model for AKI following liver transplantation and cardiac surgery by several machine learning algorithms. GBM demonstrated the most reliable performance in both investigations [75,105]. 

In the setting of cardiac surgery, Lee et al. [75] recently used machine learning techniques to predict CSA-AKI among 2010 patients undergoing cardiac surgery based on data obtained from EHR and developed an internet-based risk estimator. In comparison with logistic regression analysis, decision tree, random forest, and support vector machine displayed comparable performance with regards to AUC. GBM method exhibited the best performance with the highest AUC (C-index 0.78, compared with the logistic regression model that had a C-index of 0.69) [75]. These patterns can accurately distinguish groups of cases with different risks, and their incorporation into clinical practice can reduce intricacies and improve outcomes of CSA-AKI.

## 3. Potential Directions and Future Scope 

With the additional deepening of the investigation, machine learning-assisted monitoring may yield valuable upshots to AKI and lessen mortality and morbidity-associated CSA-AKI. The principal benefit of machine learning is in its capability to distribute with many features with multiple interactions and its specific focus on maximizing predictive performance. Nonetheless, the emphasis on data-driven prediction might dismiss mechanistic perception. Future studies are required to assess whether a machine learning model that combines AKI biomarkers (such as IL-18, NGAL, and KIM-1) [115,116,117,118] and EHR data perform better in predicting CSA-AKI than other commonly used models. Essential prerequisites are comprehensive databases with high-quality data and the evaluation and integration of AI into pragmatic clinical settings; hence, the understanding of AI and its applications in our profession is important for the present and prospective advancement of Nephrology.

## 4. Conclusions 

In summary, CSA-AKI is a complex and multifaceted syndrome associated with significant morbidity and mortality. In the present era of using big data, the application of machine learning in Nephrology clinical practice to predict AKI, including CSA-AKI, holds great future promise. 

## Figures and Tables

**Table 1:** Incidence of acute kidney injury following cardiac procedures and surgeries.

Cardiac Surgery	Incidence of AKI	Dialysis-Requiring AKI	Reference(s)
CABG (off-pump)	4.0–19.1%	2.4%	[22,23,24]
CABG (on-pump)	22.2–32.1%	1.1%	[23,24]
TAVR (mixed)	7.1–28%	1.0–2.8%	[25,26,27,28,29]
TAVR, transfemoral	18.0%	N/A	[30,31]
TAVR, transapical	38.0%	N/A	[30,31]
SAVR	12.1–29.7%	3.0–4.1%	[26,27,32,33]
MVR, surgical	19.4%	2.8%	[33]
MV repair, percutaneous	18.0%	0%	[34]
Heart transplant	47.1%	11.8%	[35]
Combined valvular surgery and CABG	4.8%	N/A	[36]
LVAD	24.9%	12.6%	[37]
Prophylactic IABP placement	5.2–10.3%	0.0–0.9%	[38,39,40]
Aortic repair, open	14.1–42.8%	N/A	[41,42]
Aortic repair, endovascular	3.7–27.1%	N/A	[41,42]
ECMO	62.8%	44.9%	[43,44]

CABG, coronary artery bypass graft; ECMO, extracorporeal membrane oxygenation; IABP, intra-aortic balloon pump; LVAD, left-ventricular assist device; MVR, mitral valve replacement; SAVR, surgical aortic valve replacement; TAVR, transcatheter aortic valve replacement.

**Table 2:** Risk predictors for acute kidney injury following cardiac procedures and surgeries from multivariate analysis.

Operative Status	Risk Factor	Subject	Odds Ratio (95% CI)	Reference(s)
*Pre-operative*	Age	CABG	1.016 (1.002–1.030)	[48]
		Cardiac surgery	4.870 (3.500–6.240)	[49]
	BMI (kg/m^2^)	SAVR	1.032 (1.007–1.057)	[50]
	Diabetes	CABG	1.360 (1.022–1.809)	[48]
		Cardiac surgery	1.520 (1.070–2.160)	[49]
	CKD	TAVR	3.530 (1.940–6.440)	[51]
	NYHA class III/IV	Cardiac surgery	2.530 (1.320–4.860)	[49]
	Hypertension	Cardiac surgery	1.680 (1.440–1.970)	[49]
	PVD	Cardiac surgery	1.310 (1.090–1.570)	[49]
	Emergency surgery	Cardiac surgery	4.760 (3.050–7.430)	[49]
*Intra-operative*	On-pump	CABG	2.630 (1.543–4.483)	[48]
	RBC transfusion	CABG	2.154 (1.237–3.753)	[48]
		SAVR	1.094 (1.006–1.191)	[50]
	CPB time	Cardiac surgery	33.780 (23.150–44.410)	[49]
	Aortic clamping time	Cardiac surgery	13.240 (7.780–18.690)	[49]
	Use of IABP	Cardiac surgery	4.440 (2.370–8.300)	[49]
*Post-operative*	Prolonged mechanical ventilation	CABG	2.697 (1.0240–7.071)	[48]
	Infection	Cardiac surgery	3.580 (1.430–8.970)	[49]
	Redo operation	Cardiac surgery	2.570 (1.750–3.780)	[49]
	Low cardiac output	Cardiac surgery	2.300 (1,050–5.040)	[49]
*Protective*	eGFR (per 10 mL/min/1.73m^2^)	TAVR	0.780 (0.680–0.870)	[25]
	Opium abuse	CABG	0.630 (0.409–0.921)	[48]

BMI, body mass index; CABG, coronary artery bypass graft; CKD, chronic kidney disease; CPB, cardiopulmonary bypass; eGFR, estimated glomerular filtration rate; IABP, intra-aortic balloon pump; NYHA, New York Heart Association; PVD, peripheral vascular disease; RBC, red blood cell; SAVR, surgical aortic valve replacement; TAVR, transcatheter aortic valve replacement.
